# Supplementary material for: A Case Study Demonstrates That the Litter of the Rare Species Cinnamomum migao Composed of Different Tissues Can Affect the Chemical Properties and Microbial Community Diversity in Topsoil
Source: Microorganisms. 2022 May 30;10(6):1125. doi: 10.3390/microorganisms10061125 (PMC9231042; doi:10.3390/microorganisms10061125)
Supplement: Supplementary file 1 [file microorganisms-10-01125-s001.zip › microorganisms-1723222-supplementary.pdf]

Table S1 The initial chemical composition of the topsoil.

pH value	TN mg·kg^{-1}	TP g·kg^{-1}	TK g·kg^{-1}	AN g·kg^{-1}	AP mg·kg^{-1}	AK mg·kg^{-1}	Catalase mL·g^{-1}·20min	Urease mgNH$_3$-N/g	Phophatase umol·g^{-1}·d^{-1}	Invertase mg·g^{-1}·d^{-1}
6.06±0.1	0.1±0.032	0.2±0.032	0.29±0.069	52.5±4.11	3.5±0.68	13.03±1.02	1.43±0.034	0.015+0.00	23.53±2.98	1.51±0.66

In one-way ANOVA of all samples, Turkey was used to compare the multiple means ($P < 0.05$), which was significant difference; there was no significant difference when the same letter was used, and there was significant difference between different letters.

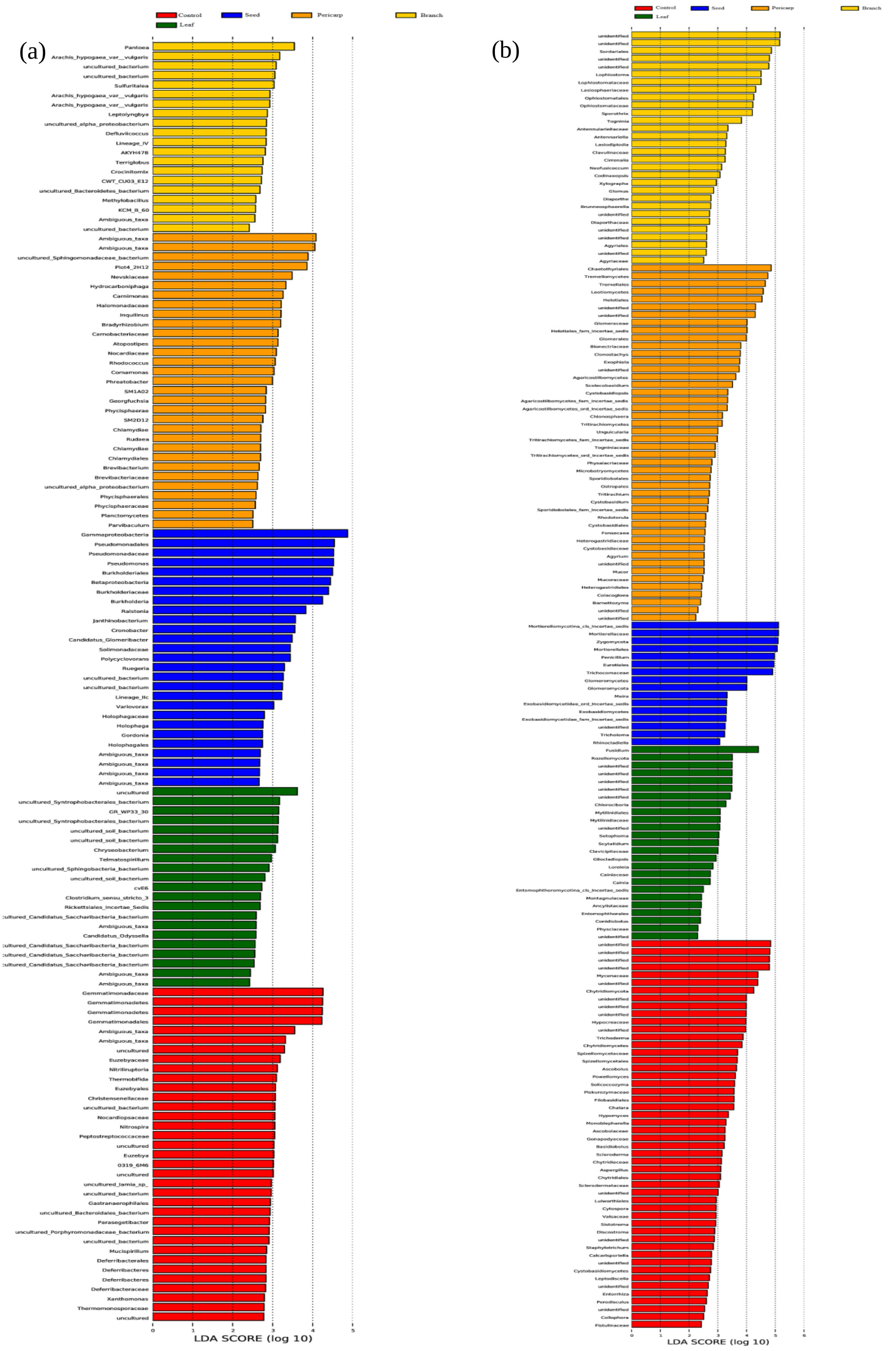

Figure S1 In the score chart of microbial differential species in different treatments. (a) fungus, (b) bacteria: The different colors represent different groups, the red bar represents the species with relatively high abundance in the red group, and the green bar represents the species with relatively high abundance in the green group.

Table S2 topological composition characteristics of network structure

different treat	Number of nodes		Number of edges		Number of neighbors		Characteristic path length		Clustering coefficient		Network density	
	fungus	bacteria	fungus	bacteria	fungus	bacteria	fungus	bacteria	fungus	bacteria	fungus	bacteria
control	222	911	6759	50233	58.89	110.28	1.60	1.84	0.49	0.49	0.13	0.08
leaf	246	316	3031	6514	22.64	39.22	1.86	1.84	0.48	0.48	0.49	0.08
branch	246	306	3031	4056	22.64	30.20	1.86	1.79	0.49	0.48	0.05	0.05
pericarp	308	1185	6556	76652	40.59	127.3	1.78	1.86	0.50	0.48	0.07	0.05
seed	271	905	3788	42675	27.56	92.30	1.85	1.87	0.49	0.49	0.05	0.05

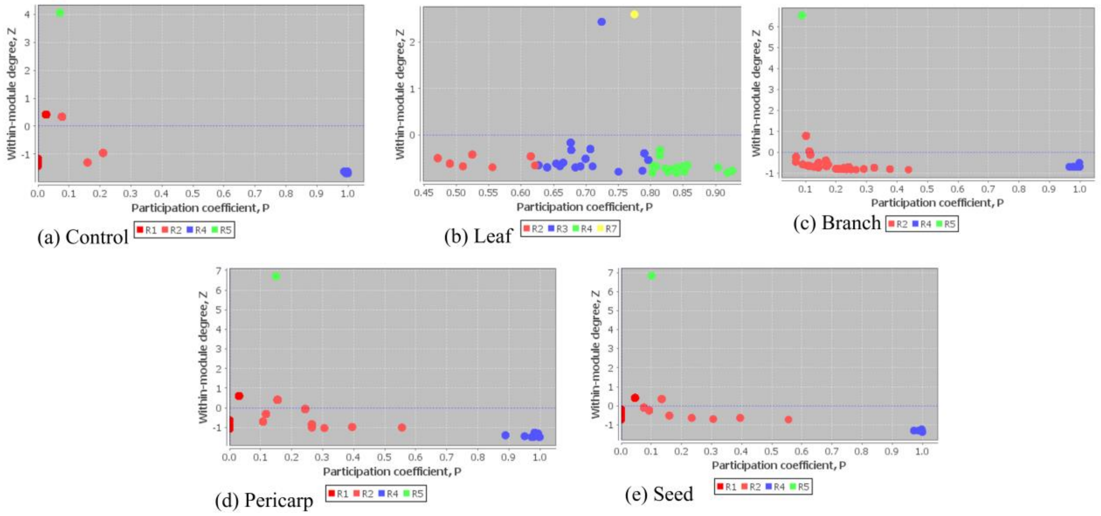

Figure S2 *Zi–Pi* plot showing the distribution of OTUs based on their topological roles in networks fungi. Each symbol represented an OTU in the bacterial (filled circle) or fungal (empty circle) network.

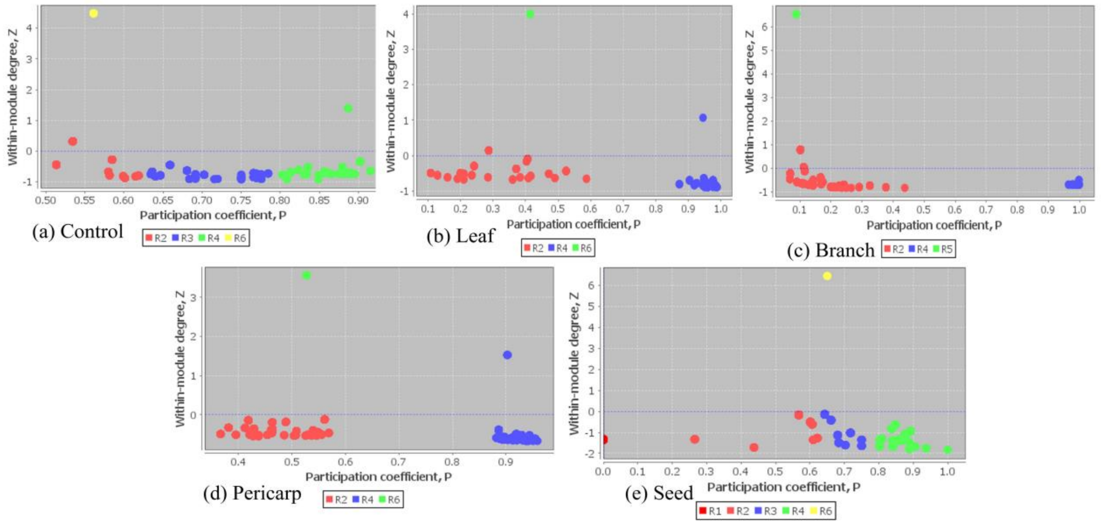

Figure S3 *Zi–Pi* plot showing the distribution of OTUs based on their topological roles in networks bacteria: Each symbol represented an OTU in the bacterial (filled circle) or fungal (empty circle) network.
